# *Fusarium* Endophthalmitis following Cataract Surgery: Successful Treatment with Intravitreal and Systemic Voriconazole

**DOI:** 10.1155/2016/4593042

**Published:** 2016-06-22

**Authors:** Paulo A. Alves da Costa Pertuiset, Juan F. Batlle Logroño

**Affiliations:** ^1^Department of Ophthalmology, Clínica Santa María, Avenida Santa María 500, Providencia, 7520378 Santiago, Chile; ^2^Department of Cornea and Refractive Surgery, Centro Laser, C/Fantino Falco 3, Naco, 10122 Santo Domingo, Dominican Republic

## Abstract

*Purpose*. To report a case of postoperative endophthalmitis caused by* Fusarium* species successfully treated with intravitreal and systemic voriconazole after treatment failure with amphotericin B.* Methods*. Clinical case report of a 60-year-old immunocompetent woman who presents with endophthalmitis of unknown origin 4 weeks after uneventful cataract extraction and IOL implantation surgery. IOL explantation, vitrectomy with capsular bag removal, vitreous aspiration for culture, and intravitreal injection of amphotericin B (5 *μ*g/0.1 mL) were performed. Diagnosis was established by culturing the vitreous aspirate on a Sabouraud agar medium and staining with lactophenol blue solution. Five days later, there was no clinical response. The decision was made to administer a single dose of intravitreal voriconazole (2.5 *μ*g/0.1 mL) and oral voriconazole (200 mg BID) for 30 days.* Results*.* Fusarium* sp. grew on culture. Treatment with local and systemic voriconazole was started after no improvement with vitrectomy, IOL explantation, and intravitreal amphotericin B. After 1 month of treatment, the infection resolved and best-corrected visual acuity was 20/25.* Conclusion*. In patients with endophthalmitis caused by* Fusarium* sp., topical and systemic voriconazole treatment should be considered in cases resistant to intravitreal amphotericin B.

## 1. Introduction

Mold endophthalmitis is usually exogenous, is rare after eye surgery, and has an onset of symptoms occurring two or more months after surgery [1]. In tropical regions, postcataract fungal endophthalmitis may present more acutely, usually within four weeks of surgery [2]. Fungal species inoculate the aqueous and then they progress posteriorly, involving the vitreous. Many cases in the literature have occurred in outbreaks [3]. Painful decreased vision with redness and hypopyon are common at presentation. To establish the diagnosis, vitreous samples should be obtained for culture. Amphotericin B is the most commonly recommended antifungal agent [1].

## 2. Case Report

This case involves a 60-year-old housewife with unremarkable systemic medical history and ocular history of an uneventful phacoemulsification in her left eye one year ago. On examination, the Snellen best-corrected visual acuity (BCVA) in both eyes was 20/20 with a manifest refraction of +1.00 SPH in the right eye and −0.25 SPH in the left eye. She had a mild nuclear sclerotic cataract in her right eye and a posterior chamber intraocular lens (IOL) implanted within the capsular bag in her left eye. Anterior segment examination in each eye was otherwise unremarkable. Intraocular pressure and fundus examination were normal. Routine preoperative complete blood count (CBC) was normal. She underwent uneventful cataract extraction surgery with intraocular lens (Alcon AcrySof IQ SN60WF) implantation on her right eye, achieving a best-corrected Snellen visual acuity of 20/20 at the 2nd postoperative week. No intracameral antibiotic injection was performed during the surgery. A fixed combination of topical 0.3% gatifloxacin and 1% prednisolone drops was prescribed 5 times a day for 15 days. At this moment she returned to her home city in northern Chile.

**Figure 1:**
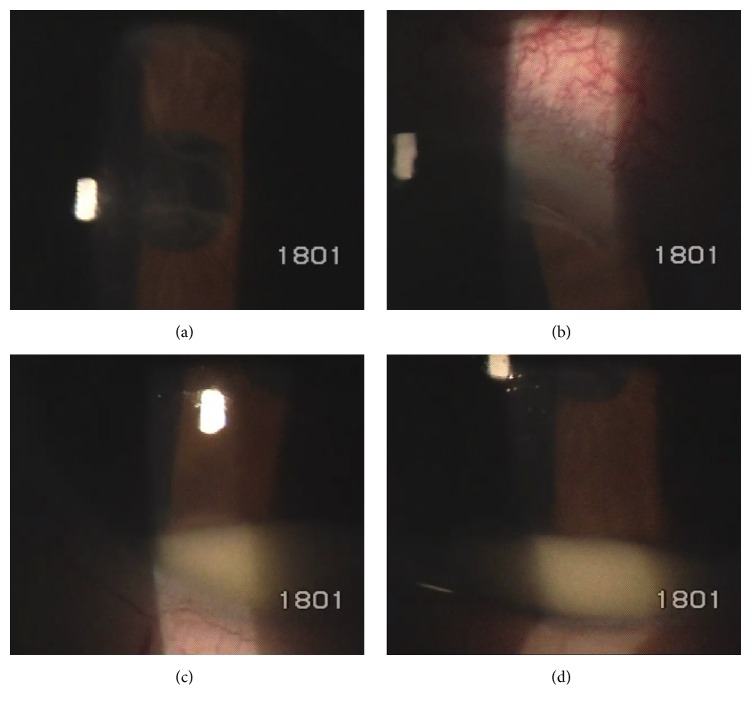
Ciliary injection, anterior chamber reaction with fibrin, and membrane formation (a and b). Hypopyon relapse (c and d).

One month after surgery, she complained of painless loss of vision with no conjunctival injection in the recently operated eye. Visual acuity dropped to 20/40. Slit-lamp examination showed hypopyon with minimal anterior chamber flare. There were no keratic precipitates, corneal edema, ciliary flush, capsular fibrosis, or anterior and posterior synechiae. Intraocular pressure in the affected eye was 14 mm Hg. Fundus examination and B-scan ocular ultrasound showed a clear vitreous. Moxifloxacin HCL 0.5% and prednisolone acetate ophthalmic suspension, USP 1% every 2 hours, were immediately started by her cataract surgeon. After three days, the hypopyon had diminished and oral Prednisone 80 mg once a day was started. This regimen was continued for 7 days, after which the patient presented with pain, corneal edema, anterior chamber reaction, ciliary injection, and an enlarged hypopyon without keratic precipitates. Repeat B-scan ultrasound showed a clear vitreous. At this moment, the patient was referred to our practice (Figure 1).

**Figure 2:**
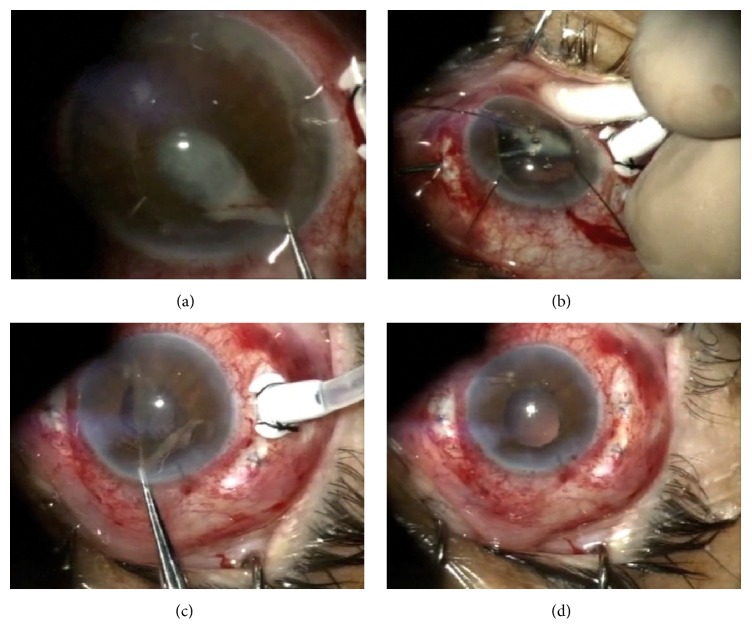
Removal of pupillary membrane (a). Shaving of vitreous base (b). Removal of iris membrane remnants (c). Concluded surgery (d).

Diagnostic vitreous culture was recommended, but the patient refused. An anterior chamber fluid sample was obtained and submitted for culture and Gram stain, but no pathogens were found. Intravitreal vancomycin (1 mg/0.1 cc) and ceftazidime (2.2 mg/0.1 mL) were injected. After one week, cultures were negative and corneal edema had intensified. Dense fibrinoid inflammatory reaction and membrane formation were noted at the pupillary border, anterior chamber, and vitreous. Given the high suspicion for fungal endophthalmitis, IOL explantation with removal of the capsular bag, vitrectomy, and cultures were performed in addition to intravitreal injection of amphotericin B (5 *μ*g/0.1 mL) (Figure 2).

**Figure 3:**
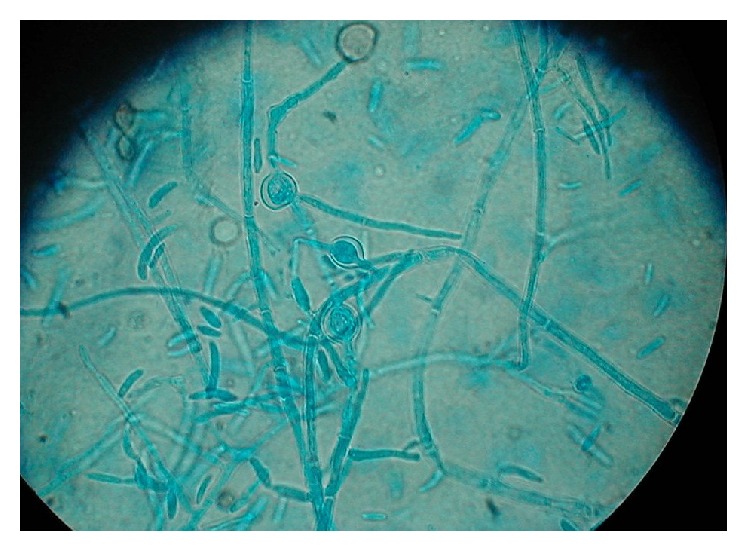
*Fusarium* sp. stained with lactophenol blue solution.

Five days later, cultures from the vitreous and explanted IOL grew* Fusarium* species (Figure 3).

**Figure 4:**
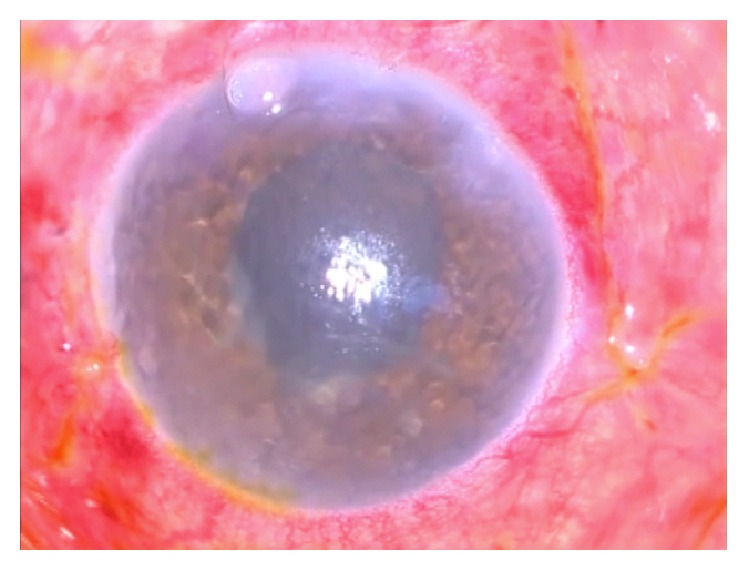
Five days after vitrectomy and intravitreal amphotericin B. Corneal edema, anterior chamber reaction, and conjunctival injection worsened.

Due to the lack of improvement (Figure 4), intravitreal voriconazole (2.5 *μ*g/0.1 mL) was administered. Oral voriconazole (200 mg BID) was also started and was continued for 30 days.

**Figure 5:**
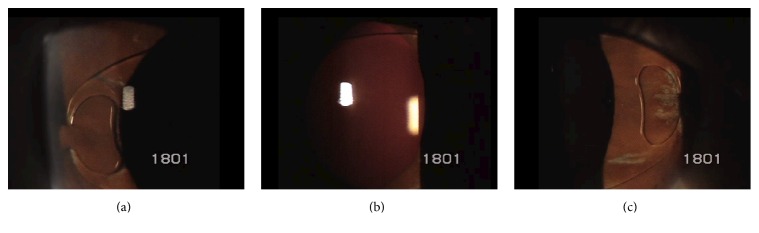
After three months Artisan phakic IOL was implanted (a to c).

After one week, the corneal edema and ciliary injection had diminished. On the same day, the microbiology laboratory informed our practice of the* Fusarium* organisms' amphotericin B resistance. One month later, there were no signs of inflammation and best-corrected visual acuity was 20/25. After three months, an Artisan phakic IOL was implanted. Following this surgery, the patient obtained a best-corrected visual acuity 20/20 with a −0.25 SPH refraction (Figure 5).

## 3. Discussion

*Fusarium* is a filamentous fungus found in the soil and on plant and vegetable matter that tends to affect mostly immunocompromised individuals [4].* Fusarium* endophthalmitis following cataract extraction surgery is a rare intraocular fungal infection. Keratitis accounts for the majority of mold exogenous endophthalmitis reported [5]. When it occurs, it is noticeably destructive because* Fusarium* species produce extracellular proteases that cause matrix degeneration. This explains the poor visual prognosis of fungal endophthalmitis [4–7].

Systemic and intravitreal amphotericin B is the most commonly used drug for the treatment of fungal endophthalmitis [8] due to its superior coverage of molds and low cost compared with other antifungal agents. However, in this case, the patient showed little improvement 5 days after its administration. Certain* Fusarium* strain resistance to amphotericin B is not uncommon, for it has been reported as one of the most drug-resistant fungi [4–7].

Intravitreal and systemic voriconazole has been reported as a safe and effective therapy for severe* Fusarium* endophthalmitis and has been used successfully as a salvage therapy in recalcitrant cases [7, 9–11]. Voriconazole is a synthetic, second-generation, broad-spectrum triazole derivative of fluconazole that inhibits the cytochrome p450 (CYP) dependent enzyme 14-alpha-sterol demethylase, required for the synthesis of ergosterol. Consequently, it disrupts the fungal cell membrane, with a broader spectrum compared to amphotericin B and fluconazole [12, 13]. Nevertheless, the high expense of voriconazole, especially oral treatment for an extended time, is a limiting factor outside the developed world. Accordingly, repeated intravitreal voriconazole injections might be more cost efficient. Unfortunately there are no controlled studies that compare intravitreal amphotericin B to voriconazole neither treatment regimen nor cumulative intravitreal toxic doses.

Postcataract surgery fungal endophthalmitis is very uncommon and usually has a delayed onset. It has to be emphasized that postcataract fungal endophthalmitis may present acutely, even within four weeks of surgery, with painless decreased vision and hypopyon as the only observable symptom and sign.

With this case report we would like to reinforce the importance of considering voriconazole in the treatment of* Fusarium* endophthalmitis, especially given the poor visual outcomes that result in the majority of cases displaying amphotericin B resistance.
